# Chronic Critical Illness in Patients with COVID-19: Characteristics and Outcome of Prolonged Intensive Care Therapy

**DOI:** 10.3390/jcm11041049

**Published:** 2022-02-17

**Authors:** Kevin Roedl, Dominik Jarczak, Olaf Boenisch, Geraldine de Heer, Christoph Burdelski, Daniel Frings, Barbara Sensen, Axel Nierhaus, Stefan Kluge, Dominic Wichmann

**Affiliations:** Department of Intensive Care Medicine, University Medical Centre Hamburg-Eppendorf, Martinistr. 52, 20246 Hamburg, Germany; k.roedl@uke.de (K.R.); d.jarczak@uke.de (D.J.); o.boenisch@uke.de (O.B.); deheer@uke.de (G.d.H.); c.burdelski@uke.de (C.B.); d.frings@uke.de (D.F.); b.sensen@uke.de (B.S.); nierhaus@uke.de (A.N.); s.kluge@uke.de (S.K.)

**Keywords:** chronic critically ill, Coronavirus Disease 2019, COVID-19, SARS-CoV-2, ICU, prolonged ICU stay, mortality, persistent critical illness

## Abstract

The spread of SARS-CoV-2 caused a worldwide healthcare threat. High critical care admission rates related to Coronavirus Disease 2019 (COVID-19) respiratory failure were observed. Medical advances helped increase the number of patients surviving the acute critical illness. However, some patients require prolonged critical care. Data on the outcome of patients with a chronic critical illness (CCI) are scarce. Single-center retrospective study including all adult critically ill patients with confirmed COVID-19 treated at the Department of Intensive Care Medicine at the University Medical Center Hamburg-Eppendorf, Germany, between 1 March 2020 and 8 August 2021. We identified 304 critically ill patients with COVID-19 during the study period. Of those, 55% (*n* = 167) had an ICU stay ≥21 days and were defined as chronic critical illness, and 45% (*n* = 137) had an ICU stay <21 days. Age, sex and BMI were distributed equally between both groups. Patients with CCI had a higher median SAPS II (CCI: 39.5 vs. no-CCI: 38 points, *p* = 0.140) and SOFA score (10 vs. 6, *p* < 0.001) on admission. Seventy-three per cent (*n* = 223) of patients required invasive mechanical ventilation (MV) (86% vs. 58%; *p* < 0.001). The median duration of MV was 30 (17–49) days and 7 (4–12) days in patients with and without CCI, respectively (*p* < 0.001). The regression analysis identified ARDS (OR 3.238, 95% CI 1.827–5.740, *p* < 0.001) and referral from another ICU (OR 2.097, 95% CI 1.203–3.654, *p* = 0.009) as factors significantly associated with new-onset of CCI. Overall, we observed an ICU mortality of 38% (*n* = 115) in the study cohort. In patients with CCI we observed an ICU mortality of 28% (*n* = 46) compared to 50% (*n* = 69) in patients without CCI (*p* < 0.001). The 90-day mortality was 28% (*n* = 46) compared to 50% (*n* = 70), respectively (*p* < 0.001). More than half of critically ill patients with COVID-19 suffer from CCI. Short and long-term survival rates in patients with CCI were high compared to patients without CCI, and prolonged therapy should not be withheld when resources permit prolonged therapy.

## 1. Introduction

Since its initial detection in December 2019, the severe acute respiratory syndrome coronavirus 2 (SARS-CoV-2) spread, causing a global healthcare emergency [1]. Up to 5% of patients with COVID-19 require admission to an intensive care unit (ICU) [2,3,4,5]. Patients at the ICU with COVID-19 suffer from high mortality, especially if invasive mechanical ventilation (MV) is necessary [2,6,7,8,9]. Prolonged ICU therapy can be commonly observed in patients with a need for MV [2,10]. Recently, it was reported that age, SOFA score, renal and vascular complications serve as independent predictors for a prolonged ICU stay [10]. Immunomodulatory therapies, including corticosteroids, IL-6 antagonists and Janus kinase 1 (JAK1) inhibitors, have recently shown beneficial effects [11,12,13,14]. However, mortality rates in critically ill patients with COVID-19 remain unacceptably high [2,15,16,17].

Overall, the management and care of critically ill patients improved substantially during the last decades owing to the constant progress of therapies and medical advances [18], leading to improved survival rates and a growing population of patients requiring ICU therapy for a longer period of time. These are also referred to as “chronically critically ill” [19,20]. Patients with a chronic critical illness (CCI) are neither recovering nor dying and are dependent on intensive care treatment due to persistent organ dysfunction [20,21,22]. Due to the large heterogeneity of this patient population, a common consensual definition does not exist. However, several studies used a cut-off of ≥21 days on ICU to define CCI [22].

Data on CCI among critically ill patients with COVID-19 is lacking, and early identification of patients at risk is needed. Further, discussion about the limitation of therapy and futility in times of overwhelmed hospitals and resource limitation is controversial, particularly in patients with a very prolonged ICU stay [20,23,24,25]. Therefore, the aim of the study was to investigate the occurrence, ICU characteristics and outcomes of patients with CCI and COVID-19.

## 2. Materials and Methods

### 2.1. Study Design, Setting and Ethics

Retrospective analysis of prospectively collected data of all consecutive patients with COVID-19 admitted to the ICUs of the Department of Intensive Care Medicine at the University Medical Centre Hamburg-Eppendorf (Hamburg, Germany) between 1 March 2020 and 8 August 2021. The department for intensive care medicine cares for all critically ill adult patients of the hospital, including 12 ICUs, with a total capacity of 140 beds. During the pandemic, a maximum of 3 ICUs were exclusively dedicated to the treatment of COVID-19 patients. The study was approved by the local clinical institutional review board and complied with the Declaration of Helsinki. The Ethics Committee of the Hamburg Chamber of Physicians was informed about the study (No.: WF-153/20). Due to the retrospective and de-identified data collection, the need for informed consent was waived.

### 2.2. Inclusion and Exclusion Criteria

We included all consecutive adult patients (≥18 years) with confirmed COVID-19 and COVID-19-associated critical illness admitted to the ICUs of our centre. Confirmed COVID-19 was defined as at least one positive result on reverse transcriptase polymerase chain reaction obtained from nasopharyngeal swabs and/or bronchial secretions. Patients with ongoing ICU treatment at the end of the study period were excluded.

### 2.3. Data Collection

Data were collected through electronic patient data management system (PDMS, Integrated Care Manager^®^ (ICM), Version 9.1-Draeger Medical, Luebeck, Germany). The extracted data included age, gender, comorbidities, admission diagnosis, length of ICU stay, treatment modalities and organ support (mechanical ventilation, vasopressor, renal replacement therapy, blood transfusions, antibiotics, antivirals, etc.) and laboratory parameters. Pre-existing medication was recorded on the basis of known regular medications and medication on admission. Routine laboratory assessment was performed on a daily basis within the usual practice.

### 2.4. Study Definitions and Patient Management

Chronic critical illness (CCI) was defined as a continuous intensive care therapy of over ≥21 days in an ICU [22]. For patients transferred from other hospitals, the first day of therapy in an ICU, including the external hospital, was counted for calculation of the length of ICU stay and consecutively for calculation of 28-/90-day mortality.

Acute respiratory distress syndrome (ARDS) was defined according to the Berlin definition, using the PaO_2_/FiO_2_ ratio (Horowitz index) as a marker for severity [26]. The severity of illness was evaluated by sequential organ failure assessment (SOFA) [27] and simplified acute physiology (SAPS II) [28] score. Charlson Comorbidity Index (CCo) [29] was calculated in all patients.

Patient and ICU management was performed following national and international recommendations, including prone positioning in severe ARDS and restrictive fluid management following the initial resuscitation period. Vasopressor support was initiated to obtain a mean arterial pressure (MAP) above 65 mmHg using norepinephrine [30,31].

Patient outcome/survival was assessed till ICU discharge and after 28-days and 90-days. The last day of follow-up was 1 November 2021.

### 2.5. Statistical Analysis

Data are presented as absolute numbers and relative frequency or median and with interquartile range (IQR). Categorical variables were compared via Chi-square analysis or Fisher’s exact test, as appropriate. Continuous variables were compared via the Mann–Whitney U test. Survival function estimates were calculated using the Kaplan–Meier method and were compared by log-rank test. We assessed factors associated with the occurrence of CCI and used a cox-regression model to assess factors associated with mortality among patients with CCI.

CCI and factors that were considered clinically relevant and did not fulfil the criteria for collinearity were included in the model. Following a stepwise-backward approach, the initial model gradually was reduced. Variables that caused a change in parameter estimates >10% or were statistically significant on a 0.05 level remained in the model. Statistical analysis was conducted using IBM SPSS Statistics Version 24.0 (IBM Corp., Armonk, NY, USA). Generally, a *p*-value < 0.05 was considered statistically significant.

The study was prepared in accordance with the Strengthening the Reporting of Observational Studies in Epidemiology recommendations.

## 3. Results

### 3.1. Study Population

**Figure 1:**
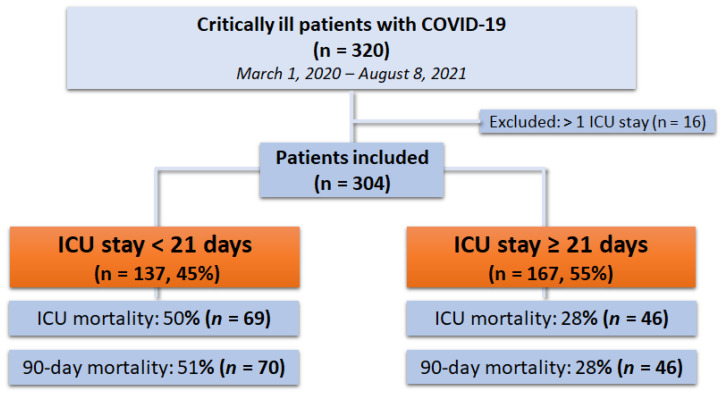
Flow chart of the study.

During the study period from 1 March 2020 to 8 August 2021, a total of 320 critically ill patients with COVID-19 were treated. After the exclusion of 16 patients with either ongoing treatment at the end of the study period or with previous ICU stay related to COVID-19, a total number of 304 critically ill patients were included in the final analysis (see study flow chart—Figure 1).

### 3.2. Baseline Characteristics of the Study Population

Detailed baseline characteristics of the study population are shown in Table 1. The median age was 61 (51–71) years, and 66% (*n* = 200) patients were male. The median Body Mass Index (BMI) was 28.1 (24.7–32.8). We observed a median Charlson Comorbidity Index (CCo) of 1 (0–3) in our cohort. Arterial hypertension (52%, *n* = 157) and diabetes mellitus (type II) (31%, *n* = 94) were the most frequent comorbidities. Seventy-eight per cent (*n* = 239) received vasopressor therapy and 46% (*n* = 140) required renal replacement therapy during the ICU stay. Overall, 73% (*n* = 223) were mechanically ventilated and 31% (*n* = 94) required extracorporeal membrane oxygenation (ECMO) therapy. The median duration of mechanical ventilation was 17 (8–38) days. Fifty-five per cent of patients (*n* = 166) were admitted from other hospitals to our tertiary care centre, the majority from other ICUs (93%, *n* = 154) or normal wards (7%, *n* = 12) of referring hospitals. Patients admitted to the ICU from our own institution were transferred from the normal ward (*n* = 88) or the emergency department (*n* = 51).

### 3.3. Occurrence, Clinical Characteristics and Complications of Patients with Chronic Critical Illness

Of the 304 critically ill patients, 55% (*n* = 167) had an ICU stay ≥21 days and were defined as chronic critical illness, and 45% (*n* = 137) had an ICU stay <21 days. Table 2 shows detailed characteristics of clinical characteristics. Age, sex and BMI were distributed equally between both groups. Patients with CCI had a numerically higher median SAPS II (CCI: 39.5 vs. no-CCI: 38 points, *p* = 0.140) and significantly higher SOFA score (10 vs. 6, *p* < 0.001) on admission and after 24 h (11 vs. 7, *p* < 0.001). Presence of ARDS was significantly more frequent in patients with (83%, *n* = 138) than without CCI (51%, *n* = 70) (*p* < 0.001). Overall, 73% (*n* = 223) patients required invasive mechanical ventilation (MV) (86% vs. 58%; *p* < 0.001). The median duration of MV was 30 (17–49) days and 7 (4–12) days in patients with and without CCI, respectively (*p* < 0.001). High-flow nasal cannula (HFNC) therapy and non-invasive ventilation (NIV) was frequently used in 46% (*n* = 140) and 42% (*n* = 128) patients overall. No significant differences regarding the use of HFNC or NIV were observed. The worst paO_2_/FiO_2_ ratio (Horowitz index) was significantly lower in patients suffering from CCI (*p* = 0.012).

Therapy strategies for treatment of moderate and severe ARDS in patients with and without CCI were prone positioning in 63% (*n* = 105) and 36% (*n* = 50) (*p* < 0.001), neuromuscular blockade in 46% (*n* = 77) and 20% (*n* = 27) (*p* < 0.001), inhaled vasodilatory treatment in 36% (*n* = 60) and 30% (*n* = 41) (*p* = 0.253) and glucocorticoid therapy in 74% (*n* = 123) and 62% (*n* = 104) (*p* = 0.717). Due to severe ARDS accompanied by life-threatening hypoxia, veno-venous extracorporeal membrane oxygenation (ECMO) was established in 38% (*n* = 64) patients with CCI and in 22% (*n* = 30) without CCI (*p* < 0.002). The rate of tracheostomy was 51% (*n* = 86) and 5% (*n* = 7), respectively. Overall, 79% (*n* = 239) patients received vasopressor support during the ICU stay, this was significantly higher in patients with CCI compared to patients without CCI (89% vs. 66%, *p* < 0.001). Renal replacement therapy was initiated in 54% (*n* = 90) with CCI and 36% (*n* = 50) of patients without CCI (*p* = 0.002), respectively. The median ICU stay of patients with CCI and without CCI was 33 (23–50) days and 7 (3–13) days, respectively.

Complications during the ICU stay were frequent, most commonly septic shock was observed in 47% (56% vs. 36%, *p* < 0.001), followed by neurologic complications in 24% (27% vs. 21%, *p* = 0.245) and cardiac arrest in 15% (17% vs. 12%, *p* = 0.176). Further deep-vein thrombosis was found in 19% (12% vs. 7%, *p* = 0.175) and pulmonary embolism was observed in 9% (8% vs. 9%, *p* = 0.756).

### 3.4. Laboratory Findings

On admission, significantly higher values of leukocytes, PCT, IL-6, Ferritin and CRP were observed in patients with CCI (all *p* < 0.05). Further, we observed a significantly higher level of D-Dimers on admission in patients with CCI compared to patients without CCI (*p* = 0.010). The median paO_2_ on admission and after 24 h was comparable between both groups. The paCO_2_ values on admission and after 24 h were significantly higher in patients with CCI; correspondingly, pH levels were significantly lower (for further laboratory results, see Supplementary Table S1).

### 3.5. Risk Factors for Chronic Critical Illness

Multivariate regression analysis identified ARDS (OR 3.238, 95% CI 1.827–5.740, *p* < 0.001) and referral from another ICU (OR 2.097, 95% CI 1.203–3.654, *p* = 0.009) as factors significantly associated with new-onset of CCI (see Table 3).

### 3.6. Outcomes and Final Discharge Destination

**Figure 2:**
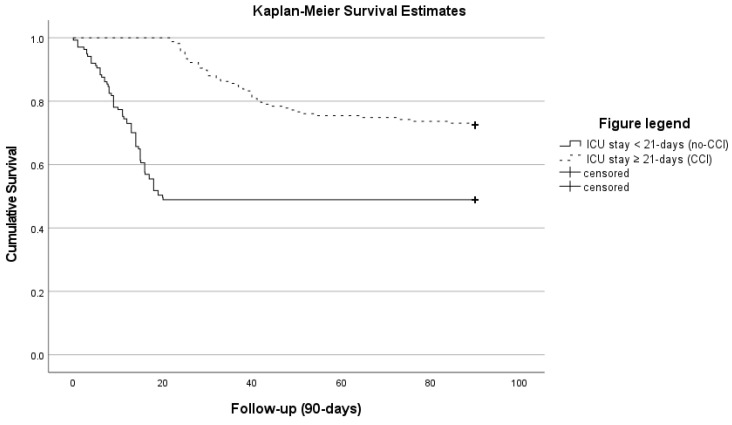
Kaplan–Meier survival estimates stratified according to patients with and without prolonged ICU stay (Log-rank: *p* < 0.001).

Overall, we observed an ICU mortality of 38% (*n* = 115) in the study cohort. In patients with CCI we observed an ICU mortality of 28% (*n* = 46) compared to 50% (*n* = 69) in patients without CCI (*p* < 0.001). The 90-day mortality was 28% (*n* = 46) compared to 50% (*n* = 70), respectively (*p* < 0.001) (see Figure 2; Kaplan–Meier survival estimates for 90-day mortality). The median length of ICU and hospital stay was 33 (23–50) and 44 (33–64) days in patients with CCI compare to 7 (3–13) and 13 (10–19) days in patients without CCI (both *p* < 0.001).

Patients with CCI who survived the ICU were discharged to normal ward in 60% (*n* = 73), specialized pulmonary rehabilitation centers in 26% (*n* = 31), other ICUs of other hospitals in 13% (*n* = 16) and to nursery care in 1% (*n* = 1). For the ICU discharge of CCI patients, 34% (*n* = 41) had a tracheal cannula in place, and 67% (*n* = 81) were successfully weaned from mechanical ventilation.

## 4. Discussion

In the present study, we investigated the occurrence of chronic critical illness in critically ill patients with COVID-19. To the best of our knowledge, this is the first study investigating the frequency, clinical characteristics and outcomes of patients with CCI in a cohort of patients with severe COVID-19. More than half of the patients developed CCI and therefore had a prolonged ICU stay. Despite the prolonged intensive care treatment, the survival rate in this population was alarmingly low. With infection rates remaining at a very high level, this underscores the need for early risk stratification to avoid unnecessary treatment and maintain medical resources.

Generally, 5–10% of patients admitted to an ICU suffer from CCI [20]. The recent literature has identified ageing and advances in critical care management as factors associated with the increasing number of patients with CCI [18,20,32]. The population of patients with a very prolonged stay at the ICU is consuming a high number of ICU resources and bed capacity [33,34,35,36]. In our investigation, we observed that most patients with CCI were invasively mechanically ventilated; the median duration of mechanical ventilation was 30 days. Furthermore, due to the high percentage of patients with severe ARDS, almost 40% of patients had to be placed on vv-ECMO due to life-threatening hypoxia. ECMO especially is a very resource-intensive therapy requiring high expertise and a high number of qualified ICU personnel and material resources. We found that more than half of the patients treated at our center suffered from CCI; the median stay at the ICU of the whole cohort was 17 days. This is in accordance with earlier studies of critically ill patients with COVID-19 [2]. However, the rate of CCI observed in our cohort was significantly higher than previously described. This could be associated with several causes. Earlier reports on CCI derived mainly from mixed-ICU cohorts, whereas our study cohort exclusively included critically ill patients with COVID-19 with a high number of high-grade respiratory failure and severe ARDS [20,37,38]. By using a cut-off of 21 days in the ICU, our cohort also included a substantial number of patients without invasive ventilation, a population not included in the aforementioned studies but remaining critically ill and dependent on intensive care therapy. Moreover, patients without mechanical ventilation and long-term ICU stay due to other organ support for the need of intensive care treatment must be included. Although this study was not designed to investigate the impact of CCI on costs or allocation of ICU resources, it seems reasonable to assume that patients with CCI require large quantities of material and personnel resources. Furthermore, differences compared to other cohorts may be attributed to different waves of the pandemic included in this study. We reported from three waves of the pandemic in Germany (#1—03/2020 to 06/2020, #2—07/2020–12/2020 and #3—01/2021–08/2021). Although we observed numerical differences in the occurrence of CCI (65% vs. 48% vs. 54%, *p* = 0.088) and mortality rates (34% vs. 48% vs. 34%, *p* = 0.059), they did not reach statistical significance. Moreover, differences in the number of referred patients (39% vs. 69% vs. 64%, *p* = 0.002) could have had an influence on the occurrence of CCI.

Chronic critical illness and prolonged mechanical ventilation are known to be associated with mortality rates of up to 50% [19,20]. Furthermore, prolonged MV or length of ICU stay are associated with unfavourable long-term survival and quality of life [38,39,40]. In patients with COVID-19, one study showed that age, SOFA score at ICU admission, paO_2_/FiO_2_, renal and cardiovascular complications were independently associated with prolonged mechanical ventilation [10]. Of interest, we observed a mortality rate of 28% among patients with CCI in our cohort, which was significantly lower than the mortality rate for COVID-19 patients without CCI (50% *p* < 0.001). Compared to reported mortality rates ranging from 40 to 42% in systematic reviews of critically ill patients with COVID-19, this appears quite low [41,42]. However, this can be a consequence of different factors. We are reporting from an experienced center in the management of ARDS and ECMO of a tertiary-care referral center. Therefore, our results may not be generally transferable to less experienced centers. Similar conclusions were reached by studies investigating the overall benefit of ECMO in ARDS and a review examining the interhospital variation in mortality among patients receiving mechanical ventilation [41,43]. We observed higher mortality in the non-CCI group. This finding is very interesting and can be related to different factors. As clearly demonstrated, patients in the non-CCI group were critically ill, and we observed similar SAPS II scores on admission; 66% required vasopressors, 36% had RRT and 58% received MV. Due to including all patients admitted during the study period, this group is very heterogenous by including also patients critically ill, but 42% did not need ventilatory support and therefore may lower the severity of the initial illness represented by the SOFA score. We, therefore, investigated the illness severity of the subgroup of patients with MV but without CCI and found that the disease severity on admission was comparable to the group of patients with CCI (SOFA median 10 pts vs. 10 pts. on admission). Furthermore, this subgroup of patients had a mortality of up to 84%, which could be related to earlier withdrawal of care due to higher severity of illness or poor overall prognosis related to comorbidities. Further, about one-third of the patients in the total cohort received ECMO therapy. This clearly demonstrates the severity of illness represented in our cohort overall. However, it is of interest that more patients in the CCI (38%) group compared to the no-CCI (22%) group received ECMO therapy. This difference can be mainly explained by the severity of illness on admission and the worst paO_2_/FiO_2_ ratio during the ICU stay, so the initiation of ECMO was either unnecessary or the patients were not suitable for ECMO due to strict selection criteria in most of the patients without CCI. Of interest, 67% of survivors with CCI were successfully weaned from mechanical ventilation, 34% had a tracheal cannula in place at discharge, and most of them were transferred to specialized pulmonary rehabilitation centers. This is in line with a recent report on outcomes after acute, long-term care in critically ill patients with COVID-19, where most patients were weaned from prolonged mechanical ventilation [44].

Discussions about the limitation of therapy and futility in critically ill patients suffering from COVID-19 are controversial, especially in times of limited resources and overwhelmed hospitals. Therefore, early planning and optimal management of resources is a cornerstone. In order to identify patients with a risk of prolonged ICU stay, we performed a logistic regression analysis. The analysis revealed that referral from an ICU from another hospital and severity of ARDS were independently associated with CCI. Referral from another ICU represents a very interesting finding; more than half of the patients in our cohort were transferred for further therapy. This may also be attributed to a strict selection process of patients eligible for advanced therapies such as ECMO or RRT. However, this study could demonstrate that a significant number of patients with CCI recovered after initial critical illness. Therefore, decisions on limitations on therapy should be based on different clinical parameters and a regular re-evaluation of the clinical situation. Length of ICU stay should not serve as a parameter limiting the therapy.

This study has several limitations. First, our study included a relatively small number of patients. Larger cohorts are needed to confirm our findings in the future. Second, our results from an experienced centre in the management of ARDS and ECMO may not be transferable to other, less experienced settings. Third, changes in clinical practice over time may have influenced outcomes of critically ill patients with COVID-19. Fourth, residual confounding is a matter of concern and cannot be entirely excluded.

## 5. Conclusions

In conclusion, we observed that more than half of the patients with severe COVID-19 suffered from CCI. Although patients required long-term intensive care, survival rates were higher than expected, and prolonged therapy should not be withheld. Further, larger studies need to confirm these results and focus on functional outcomes after CCI.

## Figures and Tables

**Table 1:** Baseline characteristics of the study cohort.

Parameters	All Patients (*n* = 304)
Demographics	
Age (years)	61 (51–71)
Sex (male)	200 (66)
Height (cm)	175 (169–180)
Weight (kg)	87 (75–100)
BMI (kg/m²)	28.1 (24.7–32.8)
Comorbidities	
Charlson Comorbidity Index (pts.)	1 (0–3)
Arterial hypertension	157 (52)
Diabetes mellitus	94 (31)
Coronary heart disease	41 (13)
Congestive heart disease	38 (13)
Chronic kidney disease	39 (13)
Chronic respiratory disease	57 (19)
Procedures during ICU	
Vasopressor therapy	239 (79)
Mechanical ventilation	223 (73)
ECMO	94 (31)
Duration of mechanical ventilation (days)	17 (8–38)
Renal Replacement Therapy	140 (46)
Admission from	
Referring Hospital	
ICU	154 (51)
Normal ward	12 (4)
Own hospital	
Emergency department	51 (17)
Normal ward	88 (29)

Data are expressed as *n* (%) or median (interquartile range). Abbreviations: BMI—Body Mass Index; pts.—points; ECMO—extracorporeal membrane oxygenation; ICU—intensive care unit.

**Table 2:** Differences between COVID-19 ICU patients with and without chronic critical illness.

Parameters	All Patients	CCI	No-CCI	*p*-Value
	(*n* = 304)	(*n* = 167)	(*n* = 137)	
Demographics				
Age (years)	61 (51–71)	61 (52–69.5)	61 (48–71)	0.494
Sex (male) (%)	200 (66)	117 (70)	83 (61)	0.083
BMI (kg/m²)	28.1 (24.7–32.8)	28.1 (24.9–33.1)	28.1 (24.3–32.7)	0.303
Disease Severity				
SAPS II (pts.)	39 (32–46)	39.5 (33–46)	38 (28.3–45.8)	0.140
SOFA—admission (pts.)	8 (4–12)	10 (5–12)	6 (3–11)	<0.001
SOFA—24 h (pts.)	9 (4–13)	11 (7–13)	7 (3–12)	<0.001
ARDS				<0.001
No ARDS	96 (32)	29 (17)	67 (49)	
Mild	4 (1)	2 (1)	2 (1)	
Moderate	29 (10)	17 (10)	12 (9)	
Severe	175 (58)	119 (71)	56 (41)	
ARDS-Management				
Prone positioning	155 (51)	105 (63)	50 (36)	<0.001
Neuromuscular blockade	104 (34)	77 (46)	27 (20)	<0.001
Inhaled vasodilatory treatment	101 (33)	60 (36)	41 (30)	0.253
Glucocorticoid therapy	227 (75)	123 (74)	104 (62)	0.717
Procedures during ICU				
Vasopressors	239 (79)	149 (89)	90 (66)	<0.001
Renal replacement therapy	140 (46)	90 (54)	50 (36)	0.002
High-flow nasal cannula	140 (46)	75 (45)	65 (47)	0.617
Non-invasive ventilation	128 (42)	75 (45)	53 (39)	0.298
Mechanical ventilation	223 (73)	144 (86)	79 (58)	<0.001
ECMO	94 (31)	64 (38)	30 (22)	0.002
Duration of mechanical ventilation (d)	17 (8–38)	30 (17–49)	7 (4–12)	<0.001
Tracheostomy	93 (31)	86 (51)	7 (5)	<0.001
Worst paO_2_/FiO_2_	70 (50–113)	62 (50–93)	83 (50–131)	0.012
Admission from				
Referring Hospital				
ICU	154 (51)	104 (62)	50 (36)	<0.001
Normal ward	12 (4)	3 (2)	9 (7)	0.003
Own hospital				
Emergency department	51 (17)	21 (13)	30 (22)	0.030
Normal ward	88 (29)	41 (25)	47 (34)	0.046
Complications-ICU stay				
Pulmonary embolism	25 (9)	13 (8)	12 (9)	0.756
Deep vein thrombosis	30 (19)	20 (12)	10 (7)	0.175
Cardiac arrest	45 (15)	29 (17)	16 (12)	0.166
Septic shock	143 (47)	94 (56)	49 (36)	<0.001
Neurologic	74 (24)	45 (27)	29 (21)	0.245
Outcome				
28-day mortality	83 (27)	13 (8)	70 (51)	<0.001
90-day mortality	116 (38)	46 (28)	70 (51)	<0.001
Discharged from ICU (alive)	189 (62)	121 (72)	68 (50)	<0.001
Duration of ICU stay (days) *	17 (6–36)	33 (23–50)	7 (3–13)	<0.001
Duration of hospital stay (days) *	30 (15–48)	44 (33–64)	13 (10–19)	<0.001

Data are expressed as *n* (%) or median (interquartile range). Abbreviations: ARDS—acute respiratory distress syndrome; SOFA—sequential organ failure assessment; SAPS II—simplified acute physiology score II; pts.—points; ECMO—extracorporeal membrane oxygenation; ICU—intensive care unit; * Including hospital and ICU days from admission in referring hospitals.

**Table 3:** Logistic regression model for factors associated with CCI; Hierarchical stepwise backward elimination of insignificant variables, change in parameter estimate >10% = confounding variable.

Logistic Regression	Covariables	OR (95% CI)	*p* Value
Final model	ARDS (yes vs. no)	3.238 (1.827–5.740)	<0.001
	Referral other ICU (yes vs. no)	2.097 (1.203–3.654)	0.009
	Age (years)	1.015 (0.998–1.033)	0.087

Abbreviations: ARDS—acute respiratory distress syndrome; CI—confidence interval; ICU—intensive care unit; OR—odds ratio.

## Data Availability

Data sharing is not applicable to this article.

## Supplementary Materials

The following are available online at https://www.mdpi.com/article/10.3390/jcm11041049/s1, Supplementary Table S1—Selected laboratory and blood gas analysis markers in COVID-19 ICU patients with and without chronic critical illness (admission and following timepoints refer to the first day of the tertiary care hospital); Supplementary Table S2—Pre-existing comorbidities of patients with severe respiratory failure at time of cardiac arrest stratified according to patients with and without COVID-19; Supplementary Table S3—Logistic regression model for factors associated with CCI; Hierarchical stepwise backward elimination of insignificant variables, change in parameter estimate >10% = confounding variable.
