# Supplementary material for: Chronic Critical Illness in Patients with COVID-19: Characteristics and Outcome of Prolonged Intensive Care Therapy
Source: J Clin Med. 2022 Feb 17;11(4):1049. doi: 10.3390/jcm11041049 (PMC8876562; doi:10.3390/jcm11041049)
Supplement: Supplementary file 1 [file jcm-11-01049-s001.zip › jcm-1577707-supplementary.pdf]

- **Supplementary Table S1** – Selected laboratory and blood gas analysis markers in COVID-19-ICU patients with and without chronic critical illness (admission and following timepoints refer to the first day of the tertiary care hospital).

- **Supplementary Table S2** – Pre-existing comorbidities of patients with severe respiratory failure at time of cardiac arrest stratified according patients with and without COVID-19.

- **Supplementary Table S3 –** Logistic regression model for factors associated with CCI; Hierarchical stepwise backwards elimination of insignificant variables, change of parameter estimate >10% = confounding variable.

Supplementary Table S1 – Selected laboratory and blood gas analysis markers in COVID-19-ICU patients with and without chronic critical illness (admission and following timepoints refer to the first day of the tertiary care hospital).

Parameters	***All patients*** (n = 304)	***CCI*** *(n = 167)*	***No-CCI*** *(n = 137)*	*p-value*
**Laboratory results**				
Haemoglobin – admission* (g/dl)	11.4 (9.7 – 12.9)	11.4 (9.6 – 12.7)	11.5 (9.8 – 13.2)	0.410
Leukocytes – admission (G/l)	9.9 (6.4 – 14.9)	10.8 (6.9 – 15.9)	8.6 (5.8 – 13.1)	0.023
Thrombocytes – admission (G/l)	236 (158 – 321)	239 (157 – 334)	218 (159 – 304)	0.268
Creatinine – admission (mg/dl)	0.97 (0.7 – 1.81)	1.04 (0.75 – 1.92)	0.87 (0.68 – 1.69)	0.110
PCT – admission (qg/l)	0.34 (0.14 – 1.38)	0.51 (0.21 – 1.72)	0.25 (0.10 – 1.28)	0.003
D-Dimer – admission (mg/l)	3.08 (1.2 – 6.7)	3.48 (1.39 – 8.48)	2.74 (0.96 – 4.65)	0.010
CRP – admission (mg/l)	154 (70 – 249)	179 (78 – 270)	133 (60 – 215)	0.005
INR – admission	1.0 (1.0 – 1.2)	1.0 (1.0 – 1.1)	1.0 (1.0 – 1.1)	0.193
IL-6 – admission (ng/l)	85 (29 – 243)	108 (37 – 276)	65 (16 – 211)	0.024
Ferritin – admission (qg/l)	1053 (594 – 2130)	1172 (680 – 2297)	937 (430 – 1920)	0.021
Bilirubin – admission (mg/dl)	0.6 (0.4 – 1)	0.6 (0.4 – 1)	0.7 (0.4 – 1)	0.533
AST – admission (U/l)	53 (36 – 88)	56 (38 – 90)	49 (33 – 86)	0.184
ALT – admission (U/l)	39 (22 – 65)	40 (26 – 68)	34 (18 – 64)	0.061
**Blood gas analysis**				
paO_2 – admission (mmHg)	74.1 (63.9 – 95.4)	73.3 (64.0 – 95.5)	76 (63.7 – 92.5)	0.867
paO_2 – 24h (mmHg)	72.0 (64.4 – 81.9)	73.4 (65.4 – 83.1)	69.3 (63.5 – 80.3)	0.066
$paCO_2$ – admission (mmHg)	41.5 (33.5 – 55.1)	45.4 (35.9 – 57.25)	37.2 (33 – 47.6)	< 0.001
$paCO_2$ – 24h (mmHg)	41.8 (35.0 – 51.0)	44.5 (37.2 – 54.3)	38.4 (33 – 46)	< 0.001
pH – admission	7.39 (7.31 – 7.47)	7.39 (7.29 – 7.46)	7.43 (7.33 – 7.48)	0.034
pH – 24h	7.42 (7.35 – 7.46)	7.41 (7.34 – 7.45)	7.44 (7.37 – 7.47)	0.002
pH – nadir	7.22 (7.10 – 7.36)	7.19 (7.11 – 7.30)	7.31 (7.09 – 7.39)	0.009
Lactate – admission (mmol/l)	1.2 (0.8 – 1.7)	1.2 (0.8 – 1.7)	1.1 (0.8 – 1.8)	0.945
Lactate – 24h (mmol/l)	1.4 (1.1 – 2.1)	1.5 (1.0 – 2.1)	1.3 (0.9 – 2.1)	0.234
Lactate – Peak (mmol/l)	3.3 (2.1 – 6.9)	3.3 (2.2 – 5.2)	3.3 (1.8 – 11.2)	0.538

Data are expressed as median (interquartile range); *admission refers to the admission at the tertiary care hospital.

Supplementary Table S2 – Pre-existing comorbidities of patients with severe respiratory failure at time of cardiac arrest stratified according patients with and without COVID-19.

Parameters	***All patients*** (n = 304)	***CCI*** *(n = 167)*	***No-CCI*** *(n = 137)*	*p-value*
**Charlson Comorbidity Index** (pts.)	1 (0 – 3)	1 (1 – 3)	1 (0 – 3)	0.456
**AIDS** *n (%)*	0 (0)	0 (0)	0 (0)	-
**Cerebral arterial disease** *n (%)*	47 (15)	23 (14)	24 (18)	0.369
**Chronic lung disease** *n (%)*	57 (19)	30 (18)	27 (20)	0.698
**Chronic kidney disease** *n (%)*	39 (13)	21 (13)	18 (13)	0.884
**Congestive heart disease** *n (%)*	38 (13)	17 (10)	21 (15)	0.177
**Connective tissue disease** *n (%)*	19 (6)	12 (7)	7 (5)	0.457
**Coronary heart disease** *n (%)*	41 (13)	19 (11)	22 (16)	0.234
**Dementia** *n (%)*	5 (2)	2 (1)	3 (2)	0.499
**Diabetes Mellitus** *n (%)*	94 (31)	60 (36)	34 (25)	0.375
**Liver cirrhosis** *n (%)*	8 (3)	3 (2)	5 (4)	0.117
**Peripheral arterial disease** *n (%)*	12 (4)	6 (4)	6 (4)	0.726
**Malignancy (Tumour; Leukamia)** *n (%)*	61 (20)	33 (20)	28 (20)	0.883

Data are expressed as n (%) or median (interquartile range); ***Abbreviations:*** AIDS, acquired immune deficiency syndrome; n, number;

Supplementary Table S3 – Logistic regression model for factors associated with CCI; Hierarchical stepwise backwards elimination of insignificant variables, change of parameter estimate >10% = confounding variable.

Logistic regression	**Covariables**	**OR (95% CI)**	***p* value**
Step 1	**Vasopressor** (yes vs. no)	1.392 (0.490 – 3.956)	0.535
	**Renal Replacement Therapy** (yes vs. no)	1.028 (0.522 – 2.026)	0.936
	**ARDS** (yes vs. no)	1.956 (0.594 – 6.449)	0.270
	**Mechanical Ventilation** (yes vs. no)	1.587 (0.417 – 6.042)	0.498
	**Septic shock** (yes vs. no)	0.955 (0.457 – 1.996)	0.903
	**Referral other ICU** (yes vs. no)	2.264 (1.152 – 4.450)	0.018
	**SOFA – admission** (pts.)	0.979 (0.906 – 1.058)	0.597
	**Age** (years)	1.012 (0.994 – 1.031)	0.178
	**Gender** (male vs. female)	0.675 (0.400 – 1.139)	0.141
Step 2	**Vasopressor** (yes vs. no)	1.398 (0.494 – 3.954)	0.528
	**ARDS** (yes vs. no)	1.957 (0.594 – 6.452)	0.270
	**Mechanical Ventilation** (yes vs. no)	1.586 (0.417 – 6.036)	0.499
	**Septic shock** (yes vs. no)	0.971 (0.519 – 1.815)	0.925
	**Referral other ICU** (yes vs. no)	2.257 (1.153 – 4.420)	0.018
	**SOFA – admission** (pts.)	0.980 (0.907 – 1.058)	0.601
	**Age** (years)	1.012 (0.994 – 1.031)	0.177
	**Gender** (male vs. female)	0.674 (0.400 – 1.136)	0.139
Step 3	**Vasopressor** (yes vs. no)	1.400 (0.495 – 3.959)	0.526
	**ARDS** (yes vs. no)	1.925 (0.615 – 6.024)	0.261
	**Mechanical Ventilation** (yes vs. no)	1.586 (0.417 – 6.038)	0.499
	**Referral other ICU** (yes vs. no)	2.262 (1.157 – 4.423)	0.017
	**SOFA – admission** (pts.)	0.979 (0.908 – 1.055)	0.577
	**Age** (years)	1.012 (0.994 – 1.031)	0.178
	**Gender** (male vs. female)	0.675 (0.401 – 1.137)	0.139
Step 4	**Vasopressor** (yes vs. no)	1.301 (0.477 – 3.552)	0.607
	**ARDS** (yes vs. no)	1.906 (0.611 – 5.943)	0.266
	**Mechanical Ventilation** (yes vs. no)	1.539 (0.408 – 5.811)	0.525
	**Referral other ICU** (yes vs. no)	2.037 (1.164 – 3.565)	0.013
	**Age** (years)	1.012 (0.994 – 1.030)	0.186
	**Gender** (male vs. female)	0.680 (0.404 – 1.144)	0.146
Step 5	**ARDS** (yes vs. no)	1.974 (0.638 – 6.106)	0.238
	**Mechanical Ventilation** (yes vs. no)	1.795 (0.544 – 5.926)	0.337
	**Referral other ICU** (yes vs. no)	1.049 (1.171 – 3.585)	0.012
	**Age** (years)	1.103 (0.996 – 1.031)	0.137
	**Gender** (male vs. female)	0.684 (0.407 – 1.151)	0.152
Step 6	**ARDS** (yes vs. no)	3.193 (1.798 – 5.670)	< 0.001
	**Referral other ICU** (yes vs. no)	2.101 (1.204 – 3.668)	0.009
	**Age** (years)	1.014 (0.996 – 1.031)	0.126
	**Gender** (male vs. female)	0.702 (0.419 – 1.177)	0.180
Final model	**ARDS** (yes vs. no)	3.238 (1.827 – 5.740)	< 0.001
	**Referral other ICU** (yes vs. no)	2.097 (1.203 – 3.654)	0.009
	**Age** (years)	1.015 (0.998 – 1.033)	0.087

Abbreviations: ARDS, acute respiratory distress syndrome; CI, confidence interval; ICU, intensive care unit; OR, odds ratio;
